# A New Method for Simultaneous Determination of Phenolic Acids, Alkaloids and Limonoids in Phellodendri Amurensis Cortex

**DOI:** 10.3390/molecules24040709

**Published:** 2019-02-15

**Authors:** Yao Chen, Zhao Zhang, Yang Zhang, Xiaomei Zhang, Zhipeng Zhang, Yonghong Liao, Bengang Zhang

**Affiliations:** 1 Institute of Medicinal Plant Development, Chinese Academy of Medicinal Sciences and Peking Union Medical College, Beijing 100193, China; chyao11@163.com (Y.C.); pdzhangyang@126.com (Y.Z.); jia5645465@163.com (Z.Z.); yhliao@implad.ac.cn (Y.L.); bgzhang@implad.ac.cn (B.Z.); 2 China-ASEAN Traditional Medicine Exchange and Cooperation Center, Guangxi Botanical Garden of Medicinal Plants, Nanning 530023, China; zhangxiaomei_2016@163.com

**Keywords:** phellodendri amurensis cortex, phenolic acids, alkaloids, limonoids, multi-wavelength, quality evaluation

## Abstract

Phellodendri Amurensis Cortex (PAC) is a well-known herbal medicine in China with complex components, but the previous research has mostly focused on its alkaloids analysis. For the first time, a simpler and more efficient method was proposed in this paper to simultaneously determine the content of three different kinds of compounds—phenolic acids, alkaloids and limonoids—in PAC. The phenolic acids included 3-*O*-feruloylquinic acid, 4-*O*-feruloylquinic acid and syringin. The alkaloids include magnoflorine, phellodendrine, jatrorrhizine, palmatine and berberine, while the limonoids include obaculactone and obacunone. An approach combining multi-wavelength and HPLC-DAD was used in this study due to the great difference in maximum absorption wavelength of the various components. Four wavelengths at 215, 275, 280 and 310 nm, respectively, were chosen for monitoring. It has been indicated through appropriate tests that this approach is of high accuracy, good repeatability and stability and provides a scientific basis for the quality assessment of PAC and associated derivatives. In addition, the chromatographic fingerprints method combined with multivariate statistical analysis chosen in this study was proved to be effective and reasonable for an accurate classification of 33 batches of samples collected from different locations.

## 1. Introduction

Phellodendri Amurensis Cortex (PAC), known as “Guan Huang Bai” in China, is derived from the dried bark of *Phellodendron amurense* Rupr. and mainly distributed in the northeastern region of China [1]. In recent years, in addition to the traditional efficacy of clearing heat and eliminating dampness, detoxifying and eliminating inflammation; further biological activity has been discovered in modern pharmacological studies of PAC, including antioxidant, hypoglycemic, antitumor activity, induced specific immune tolerance, neuroprotection and so on [2,3,4,5,6,7]. For centuries, PAC has been utilized solely or in combination with other medicines for the clinical treatment of various diseases, and used to produce drugs such as Huangbai capsule, Zhi-bai-di-huang pills, Huang-lian-jie-du decoction, Er-miao pills, Er-xian decoction, Si-miao pills, etc. [8,9,10,11].

Phytochemical studies in the literature show that PAC was known to contain a complex mixture of ingredients, including alkaloids, phenolics, limonoids and so on [12,13,14]. Most studies have reported on the biological activities of the alkaloids derived from PAC, including their antimicrobial activity [15], anti-inflammation activity [16], and neuroprotective activity [5,17]. Phenolic acids are also a widely distributed group of secondary metabolites that possess strong anti-oxidative activities, and they are generally added into medicinew to prevent cancer and bacterial cell growth [18,19]. Limonoids show protective effects against glutamate-induced neurotoxicity in primary cultures of rat cortical cells [15,20]. Thus it can be seen PAC is a medicinal plant with high medicinal value, showing strong and far-ranging biological and pharmacological activity, which makes is worthy of further research and development.

**Figure 1:**
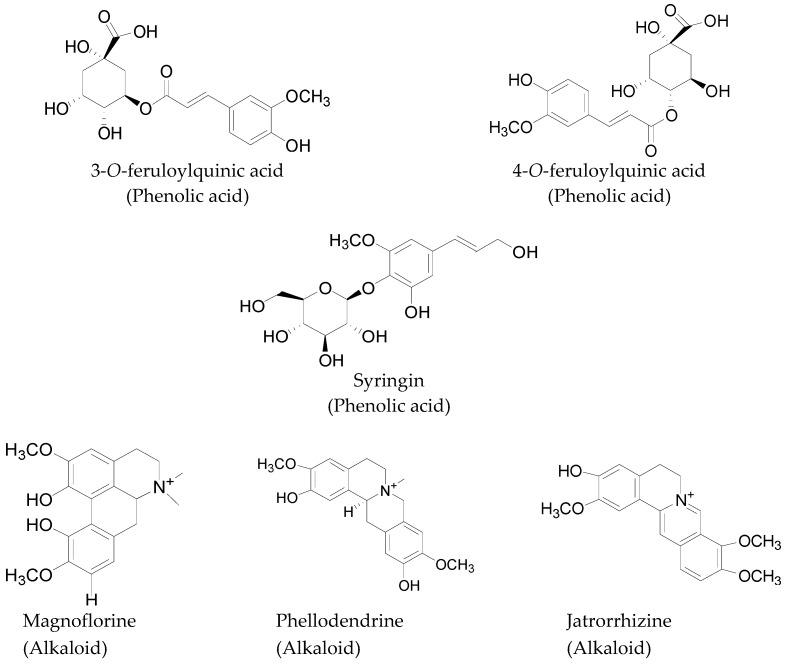

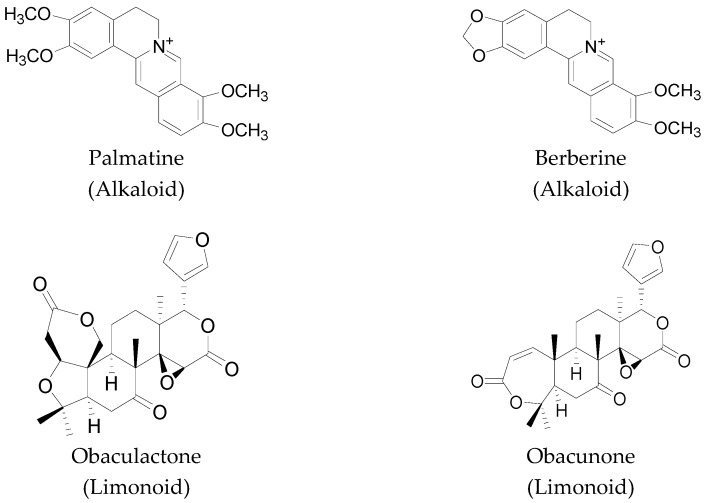
Structures of the 10 constituents identified in Phellodendri Amurensis Cortex (PAC).

Considering the complexity of herbal medicines, slight differences in various components may greatly affect the therapeutic effects [21,22]. In accordance with the Chinese Pharmacopoeia, palmatine and berberine were used as the chemical indicators of PAC when the pharmacodynamics function was not yet clear. The current quality control of PAC is insufficient, as most of the reports merely focus on the qualitative and quantitative analysis of its alkaloids [23,24,25,26]. Although several analytical methods based on HPLC, ^1^H-NMR, HPLC-DAD-MS have been employed to quantify chemical markers, they have simultaneously determined only same type of compound [23,24,25,26]. Few studies so far have reported on the simultaneous determination of the content of the multiple bioactive components of PAC, making a rapid and validated multicomponent analytical method still highly desirable for the systematic evaluation of its quality. HPLC-DAD-ESI-MS/MS has been used for the systematic characterization and identification of chemical constituents, and multi-component quantification. Therefore, in present study, we attempted to establish a comprehensive and systematical method that combines qualitative assessment and quantitative analysis of phenolic acids, alkaloids and limonoids for quality control of PAC. The objectives of this study were thus to develop an improved HPLC-DAD-ESI-MS/MS method for the simultaneous separation, identification and quantitation of ten phenolic acids, alkaloids and limonoids in PAC extract (Figure 1).

## 2. Results and Discussion

### 2.1. Identification of Constituents

The identity of the various chromatographic peaks was identified by comparing their retention time, accurate mass and MS/MS fragmentation patterns with the data reported in the literature or commercial standards. Considering the complexity of the chemical constituents in PAC, both the positive and negative ion modes were employed for the identification. The mass spectra data of compounds is shown in Table 1. Since the identification of alkaloids in Phellodendri Amurensis Cortex has been elaborated in a lot of literature [23], the authors will not repeat it in this paper.

#### 2.1.1. Characteristics of the Phenolic Constituents

**Scheme 1:**
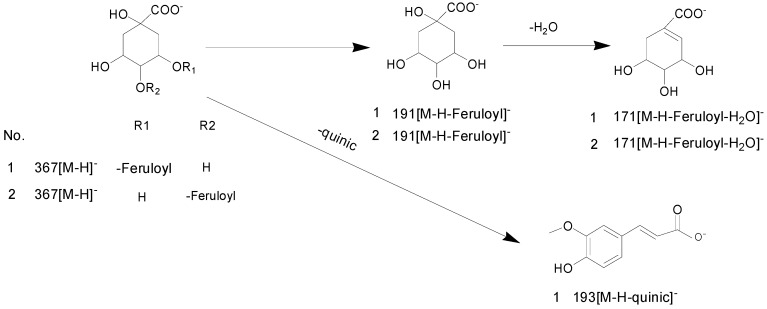
Proposed fragmentations of chlorogenic acid, 3-*O*-feruloylquinic acid (1) and 4-*O*-feruloylquinic acid (2).

**Scheme 2:**
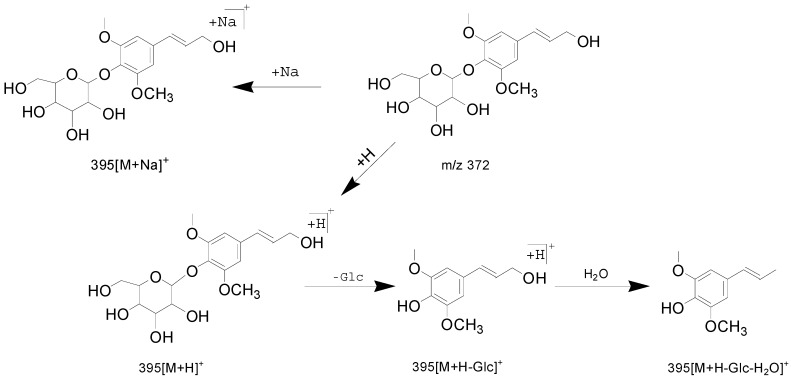
Proposed fragmentation of syringin.

For the three phenolics negative ion mode because of its clearer fragmentation pattern for structural identification of most of the compounds, 3-*O*-feruloylquinic acid and 4-*O*-feruloylquinic acid, whereas the response of syringin in positive mode was much higher than that in negative mode. The identities were corroborated with the fragmentation patterns derived from their mass spectra. 3-*O*-Feruloylquinic acid and 4-*O*-feruloylquinic acid presented different retention times (9.64 min and 10.35 min, respectively), but they both showed the same profile, with peaks at *m*/*z* 367,191,193,173, and the identities were eventually confirmed by comparison with their respective commercial standards. The *m*/*z* 367 peak corresponds to the [M − H]^−^, while the fragment ions at *m*/*z* 191, 173 and 193 correspond to [M − H-feruloyl]^−^, [M − H-feruloyl-H_2_O]^−^, and [M − H-quinic]^−^, respectively. Syringin showed a fragmentation pattern with four characteristic peaks, among which the molecular ion (*m*/*z* 373 [M + H]^+^) displayed a relatively higher abundance compared to the others; in addition, fragment ions at *m*/*z* 395 [M+Na]^+^, 211 [M + H-Glc]^+^,193 [M + H-Glc-H_2_O]^+^ were also observed. The fragmentation schemes of these phenolics are shown in Scheme 1 and Scheme 2.

#### 2.1.2. Characteristics of the Limonoids

**Scheme 3:**
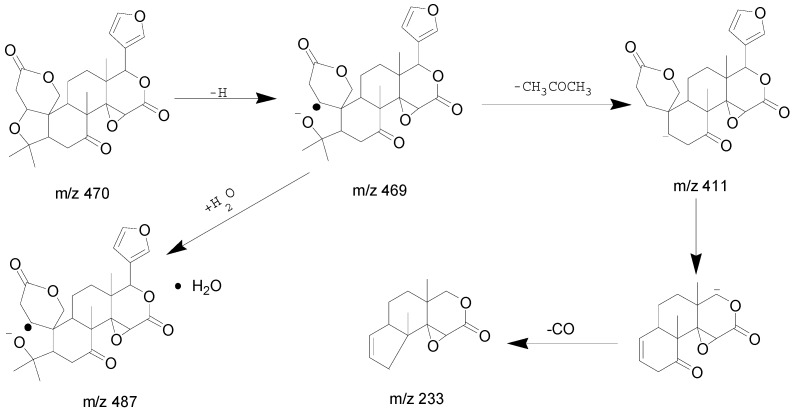
Proposed fragmentation of obaculactone.

Two limonoids were detected in both positive and negative modes, and respectively identified as obaculactone and obacunone. In positive ion mode, obaculactone and obacunone produced [M + H]^+^ molecular ions at *m*/*z* 471 and 455, respectively. In negative ion mode, both of them appeared as the deprotonated [M − H]^−^ ion at *m*/*z* 469 and 453. In addition, obacunone produced fragments at *m*/*z* 411 [M − H-C_3_H_6_O]^+^, 233 [M − H-C_3_H_6_O-C_8_H_6_O_3_-CO]^+^ and 487 [M − H + H_2_O]^+^ as major ion peaks. The proposed fragmentation pathway in negative mode of obaculactone is illustrated in Scheme 3.

### 2.2. Optimization of Sample Preparation

Some factors influencing the extraction efficiency of the target analytes were optimized for more efficient extraction. Compared with refluxing, and soaking at room temperature, ultrasonic extraction was simpler and more effective. Subsequently other experimental factors were optimized as follows: different extraction solvents (1% hydrochloric acid within methanol, 70% methanol, methanol), different sample-solvent ratios (0.5:30, 0.5:40, 0.5:50 *w*/*v*), different extraction time (10, 20, and 30 min) and extraction cycles (one, two or three cycles). Comparing the number, areas and resolution of the chromatographic peaks obtained by the different extraction procedures, the optimized extraction conditions were determined as ultrasonic extraction with 70% methanol of a sample-solvent ratio 1:40 (*w*/*v*), for three times (20 min each).

### 2.3. Optimization of the HPLC-DAD Method

To obtain optimal chromatographic conditions with a good separation of as many peaks as possible in a short analysis time, different HPLC parameters including column types, mobile phase composition, gradient elution profile, flow rate of the mobile phase and column temperature were examined and compared. Thus, various columns Waters XBridge C18 (Waters Corporation, Milford, MA, USA), 250 mm × 4.6 mm, 5 μm; Agilent Zorbax SB-C18 (Agilent Corporation, Santa Clara, CA, USA), 250 mm × 4.6 mm, 5 μm; Agilent Eclipse XDB C18 (Agilent Corporation)250 mm × 4.6 mm, 5 μm), mobile phases (acetonitrile–water and methanol–water with different modifiers, including formic acid, acetic acid, phosphoric acid and ammonium bicarbonate), column temperatures (25 °C, 30 °C, 35 °C and 40 °C), and mobile phase flow rates (0.8, 1.0 and 1.2 mL/min) were evaluated, respectively.

Generally, the maximum UV absorption wavelength of target analytes is often chosen as the detection wavelength. However, the differences in structural features and properties resulted in different UV absorption profiles, and a single-wavelength-based HPLC fingerprint no longer represents the overall characteristics. Multi-wavelength detection in combination with HPLC may be a useful strategy for the analysis of complex constituents with different maximum absorptions, which and is widely used for herbal medicines [27,28,29]. In PAC, the peak signals of phenolic acids were relatively strong with 310 nm wavelength detection, whereas alkaloids showed better peaks at 275 nm or 280 nm and limonoids were detected at 215 nm. The difference among the three types of compound was thus huge. The Agilent 1260 Infinity Multiple Wavelength detector (MWD) (Agilent Corporation, Santa Clara, CA, USA) provides simultaneous detection of up to eight specific wavelengths for optimum selectivity within a wavelength ranges from 190 nm to 950 nm. In the present experiments, based on the elution time and the maximum UV absorption wavelength of the 10 chemical components, four wavelengths were selected for this method: 0–13 min, 310 nm; 13–17 min, 275 nm; 17–41 min, 280 nm; 41–55 min, 215 nm.

**Figure 2:**
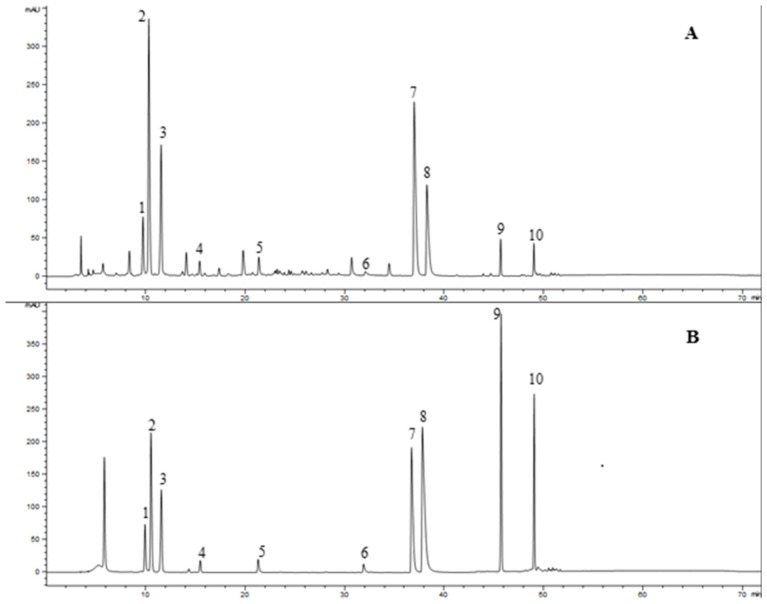
Chromatograms for chemical analysis. (**A**) Phellodendri Amurensis Cortex (PAC) sample, (**B**) standard mixture. Peak identities: 3-*O*-feruloylquinic acid (**1**), 4-*O*-feruloylquinic acid (**2**), magnoflorine (**3**), syringing (**4**), phellodendrine (**5**), jatrorrhizine (**6**), berberine (**7**), palmatine (**8**), obaculactone (**9**), obacunone (**10**).

As a consequence, optimized HPLC conditions were developed as follows by comprehensively comparing the resolution, baseline, elution time and numbers of characteristic peaks in each chromatogram of different parameters: Waters XBridge C18 column, column temperature at 25 °C, mobile phase was acetonitrile-10 mmol ammonium bicarbonate, mobile phase flow rate was 0.8 mL/min and injection volume was 5 μL, and four detection wavelengths. Figure 2 shows typical chromatograms of a mixed solution of 10 chemical standards and a PAC sample extract, respectively.

### 2.4. Validation of Quantitative HPLC-DAD Method

The calibration curves were constructed by five concentration assays with each standard in triplicate. Regression equations, the correlation coefficients (R^2^), detection and quantification limits are listed in Table 2. The high correlation coefficients (R^2^ > 0.9984) showed good linearity correlations over relatively wide concentration ranges between the concentrations and the peak areas for 10 components. The LOD and LOQ were expressed by 3- and 10-fold of signal-to-noise (S/N) ratios. The intra-day precision and inter-day precision determinations were performed on samples and standard solutions, respectively. The results showed that the RSDs of the 10 components were less than 1.49% for intra-day precision, and less than 2.02% for inter-day precision. To evaluate the repeatability and stability, six prepared samples were analyzed for validation, the low RSD values obtained for all 10 components confirmed the high repeatability and good stability (Table 3). The recovery test was carried out to further evaluate the accuracy of the method. The validation results indicated that the developed method was efficient, accurate and sensitive for multi-ingredients determination of PAC.

### 2.5. HPLC Fingerprints of PAC

**Figure 3:**
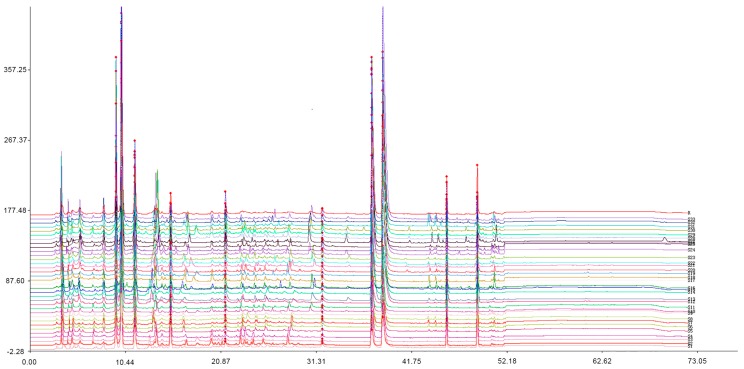
Chromatographic fingerprints for Phellodendri Amurensis Cortex (PAC) samples.

Reference chromatographic fingerprints for PAC extracts are illustrated in Figure 3. In order to compare the similarity between individual chromatograms of the studied samples and the reference fingerprint, the correlation coefficients were calculated and are listed in Table 4. Except for three samples (numbers 2, 3, 8), the similarity values for the majority of the samples varied over the range of 0.933 to 0.997, which meant that 33 batches of PAC samples showed good chemical constituent similarity.

### 2.6. Analysis of PAC Samples

The developed and validated HPLC-DAD method was applied for the determination of 10 compounds of PAC samples from different locations. The contents were calculated by the external standard method from three parallel determinations of each sample. As shown in Table 4, the content of the 10 components showed a remarkable difference in the 33 samples studied. In PAC samples, the compounds 4-*O*-feruloylquinic acid (3.347–29.666 mg/g), berberine (1.935–25.745 mg/g) and magnoflorine (1.470–21.721 mg/g) were present in the highest content, followed by 3-*O*-feruloyl-quinic acid (1.135–16.227 mg/g), obacunone (2.352–13.042 mg/g), and palmatine (0.620–12.756 mg/g) while the contents of other ingredients were lower. These results showed that there were significant differences in the contents of 10 components among those plots. Therefore, detection of a single or only a few compounds could not control the quality of PAC effectively. *P. amurense* Rupr. is widely distributed in China and the diverse geographical sources that have different ecological environments and other factors could possibly result in great variations in their chemical constituents. Besides, the concentrations of compounds varied significantly among PAC samples from different provinces. The average content of 3-O-feruloylquinic acid from Liaoning was 7.727 mg/g, which is 2.97 times that from Heilongjiang (2.600 mg/g); the average content of 4-*O*-feruloylquinic acid from Liaoning was 22.093 mg/g, which is 3.21 times that from Heilongjiang (6.871 mg/g); the average content of magnoflorine from Liaoning was 15.362 mg/g, which is 2.51 times that from Heilongjiang (6.104 mg/g); the average content of phellodendrine from Liaoning was 2.951 mg/g, which is 2.84 times that from Heilongjiang (1.038 mg/g); the average content of palmatine from Liaoning was 8.451 mg/g, which is 2.57 times that from Heilongjiang (3.279 mg/g); the average content of berberine from Liaoning was 15.876 mg/g, which is 3.88 times that from Heilongjiang (4.090 mg/g); the average content of obaculactone from Jilin was 9.901 mg/g, which is 2.05 times that from Liaoning (4.829 mg/g); the average content of obacunone from Jilin was 2.962 mg/g, which is 2.17 times that from Liaoning (1.361 mg/g).

It can be clearly seen that the content distribution of phenolic acids and alkaloids were gradually decreased from south to north; conversely, the distribution of obaculactone and obacunone were both increased gradually from south to north. These results suggest that the source greatly affected the quality of PAC. Thus, appropriate geographical origins of herbs should be selected to ensure the safety and effectiveness of PAC products used for therapeutic purposes.

**Figure 4:**
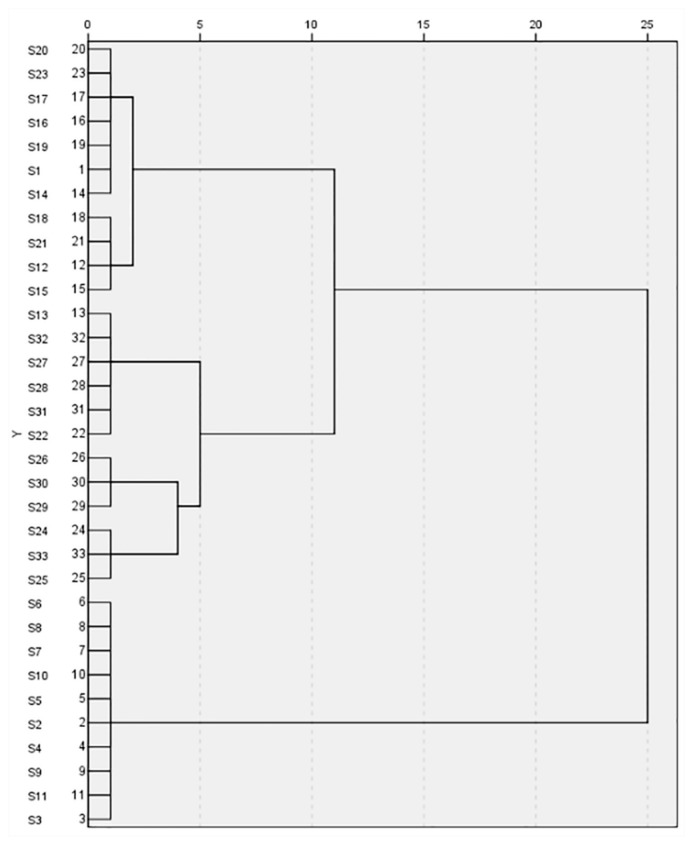
Dendrogram of hierarchical cluster analysis for the 33 Phellodendri Amurensis Cortex (PAC) samples.

Hierarchical clustering analysis (HCA) of 33 PAC samples was performed. The results are shown in Figure 4. It was clear that the samples could be divided into three clusters based on the geographical location when the Euclidean distance was between 5 and 10. The first group contained S1, S12, S14, S15, S16, S17, S18, S19, S20, S21, S23, mainly derived from Jilin, except S1 which was from Heilongjiang; the second group contained S13, S22, S24, S25, S26, S27, S28, S29, S30, S31, S32, S33, mainly derived from Liaoning except S13 and S22 from Jilin; the third group contained S2, S3, S4, S5, S6, S7, S8, S9, S10, S11, all derived from Heilongjiang. The different climates and environmental affect the formation and accumulation of phytochemicals. In addition, extraction of characteristic chemical components has an impact on the HCA results, which showed that samples from near regions were easier to cluster, which was an objective of this work, and it means that the method of HCA analysis with ten chemical components (three chemical type categories) was trustworthy.

## 3. Conclusions

The identification and quality control of herbal medicines is regularly performed by qualitative and quantitative analysis of marker compounds. This study established a multi-wavelength chromatographic fingerprints method, which better represented the complex chemical composition of PAC samples. In this paper, chemical profiling of PAC were examined by an effective HPLC-DAD-ESI-MS/MS method and 10 common constituents including phenolic acids, alkaloids and limonoids were identified based on their mass and fragmentation patterns. It provided a reliable method for quantitative analysis of these 10 components in 33 batches of PAC samples collected from different locations. Multiple components were deemed the main chemical constituents according to the HCA results and could serve as suitable markers for quality control of PAC. The results showed that analytical method established in this study could be a simple and powerful tool which provides full-scale qualitative and quantitative information for evaluating the quality of PAC, to solve the problem of insufficient quality control of PAC with only the analysis of alkaloids. It would also be a valuable reference for the further study and development of other herbal pharmaceutical products.

## 4. Materials and Methods

### 4.1. Plant Materials and Regents

In this study, all 33 PAC samples were collected from natural distribution areas of wild *Phellodendron amurense* Rupr., which were typical and representative sample plots in Heilongjiang, Jilin, and Liaoning (Table 5). The samples were collected from 2013 to 2015, then air-dried and stored at −38 °C.

Acetonitrile (HPLC grade) was purchased from Merck KGaA (Darmstadt, Germany). Pure water (18.2 M) was purified by Milli-Q system (Millipore, Billerica, MA, USA) and used to prepare buffer and sample solutions. Analytical grade methanol was purchased from Beijing Chemical Works (Beijing, China). Ammonium bicarbonate (HPLC grade) was purchased from Mreda Technology Inc. (Fair lawn, NJ, USA). The reference standard compounds of magnoflorine, phellodendrine, jatrorrhizine, palmatine, berberine, obaculactone and obacunone (purity >98%) were purchased from Phytomarker Ltd. (Tianjin, China), while 3-*O*-feruloylquinic acid, 4-*O*-feruloylquinic acid and syringin (purity >98%) references were purchased from Wuhan ChemFaces Biochemical Co, Ltd. (Wuhan, China).

### 4.2. Preparation of Standard and Sample Solutions

Magnoflorine, syringin, phellodendrine, jatrorrhizine, palmatine, berberine, obaculactone and obacunone were accurately weighed and dissolved in methanol, while 3-*O*-feruloylquinic acid and 4-*O*-feruloylquinic acid were dissolved in 50% methanol–water (*v*/*v*) to obtain 10 reference standard compound stock solutions, respectively. After that, these solutions were diluted to within appropriate concentration ranges for the establishment of calibration curves and to prepare a mixed standard solution. PAC samples were oven-dried, ground and sieved (60-mesh), respectively. The powdered sample (0.5000 g) was weighed accurately, 3 portions were used for each kind of sample, placed in 40 mL 70% methanol with conical flask, then extracted under ultrasonic bath (80 kHZ, 250 W) thrice (20 min each) at room temperature. After cooling, it should be weighed again for compensating the weight loss with extraction solvent. The sample solution was filtered through a 0.22 μm membrane filter prior to injection into the HPLC system. All the solutions were stored in brown bottles at 4 °C.

### 4.3. HPLC-DAD Analysis

Chromatographic analysis was performed on an Agilent 1260 HPLC system (Agilent Technologies, Palo Alto, CA, USA), which consisted of a vacuum degasser, a quaternary pump, an autosampler, a thermostated column compartment and a diode array detector (DAD). Chromatographic data was processed using an Agilent Chemstation. The separation was achieved through the use of a Waters XBridge C18 column (4.6 mm × 250 mm i.d.,5 μm, Waters Corporation, Milford, MA, USA) with a mobile phase composed of (A) 10mmol NH_4_HCO_3_ in water and (B) ACN. The gradient condition was: 0–13 min, 6–12% B; 13–38 min, 12–40% B; 38–50 min, 40–75% B; 50–55 min, 75–90% B; 55–65 min, 90% B; 65–72 min 90–6% B. Each run was followed by an equilibration period of 8 min with the initial conditions (94% A, 6% B). The injection volume was 5 μL. The flow rate was 0.8 mL/min. The column and autosampler were maintained at 25 °C. The variable wavelength detector was set as follow: 0–13 min, 310 nm; 13–17 min, 275 nm; 17–41 min, 280 nm; 41–55 min, 215 nm.

### 4.4. HPLC-ESI-MS/MS Method

HPLC-ESI-MS analysis was carried out with an AB Sciex 4500 Q-Trap mass spectrometer (Applied Biosystems, Foster City, CA, USA) connecting to an Agilent 1200 HPLC system via an electro-spray ionization interface. The chromatographic conditions were described as follows. Electro-spray ionization was applied in negative ion mode for MS and MS/MS with an ion spray voltage of 4000 V, curtain gas at 30 psi, ion source gas1 and gas 2 both at 60 psi. The ion source temperature was set at 400 °C. Ultrapure nitrogen was used as the nebulizer, heater, curtain and collision-activated dissociation (CAD) gas. Data was processed using Analyst 1.4 software (Applied Biosystems, Waltham, MA, USA). MS data, retention time and UV-Vis spectra were used to identify the bioactive compounds contained in PAC. The assignments were validated by co-elution with the corresponding reference compounds and through the comparison with published data.

### 4.5. Method Validation

After the establishment of optimal conditions, method validation was performed to ensure the newly developed fingerprint identification method be monitored and evaluated effectively. Calibration curves were constructed by plotting the peak area and the concentration of the corresponding working standard solution. The analytical method was validated with respect to the limit of detection (LOD) and limit of detection quantification (LOQ), precision, repeatability, stability and recovery, respectively. Hierarchical clustering analysis (HCA) is one of the most widely used techniques for grouping multivariate data into a tree of cluster data from the 33 batches of PAC samples collected from various regions in China were subjected to cluster analysis using the SPSS 21.0 software (IBM Corporation, Armonk, NY, USA).

## Figures and Tables

**Table 1:** Identification of 10 consitutents in the extract solution of Phellodendri Amurensis Cortex (PAC) by HPLC–ESI–MS.

Type	Constituent	RT (min)	Ion Mode	MS (*m*/*z*)	MW	MS/MS Data (*m*/*z*)	Pathway
Phenolics	3-*O*-feruloyl-quinic acid	9.64	−	367 [M − H]^−^	368	191	[M-H-Feruloyl]^−^
						171	[M-H-Feruloyl-H_2_O]^−^
						193	[M-H-quinic]^−^
	4-*O*-feruloyl-quinic acid	10.35	−	366 [M − H]^−^	368	191	[M-H-Feruloyl]^−^
						171	[M-H-Feruloyl-H_2_O]^−^
	Syringin	15.64	+	373 [M + H]^+^	372	395	[M+Na]^+^
						211	[M+H-glc]^+^
						193	[M+H-glc-H_2_O]^+^
Alkaloids	Magnoflorine	11.57	+	342 [M]^+^	342	343	[M+H]^+^
						297	[M-(CH_3_)_2_NH]^+^
						279	[M-(CH_3_)_2_NH-H_2_O]^+^
						266	[M-(CH_3_)_2_NH-CH_3_OH+H]^+^
						685	[2M+H]^+^
	Phellodendrine	21.52	+	342 [M]^+^	342	343	[M+H]^+^
						327	[M-CH_3_]^+^
						192	[M-C_9_H_10_O_2_]^+^ (RDA)
						177	[M-C_9_H_10_O_2_-CH_3_]^+^
	Jatrorrhizine	32.23	+	338 [M]^+^	338	339	[M+H]^+^
						323	[M-CH_3_]^+^
						308	[M-2CH_3_]^+^
	Palmatine	37.29	+	352 [M]^+^	352	337	[M-CH_3_]^+^
						322	[M-2CH_3_]^+^
						338	[M+H-CH_3_]^+^
	Berberine	38.42	+	336 [M]^+^	336	321	[M-CH_3_]^+^
						293	[M-CH_3_-CO]^+^
						279	[M-CH_3_-CO-CH_2_]^+^
						673	[2M+H]^+^
Limonoids	Obaculactone	45.61	−	469 [M − H]^+^	470	411	[M-H-C_3_H_6_O]^+^
						233	[M-H-C_3_H_6_O-C_8_H_6_O_3_-CO]^+^
						487	[M-H+H_2_O]^+^
	Obacunone	49.08	−	453 [M − H]^+^	454	471	[M-H+H_2_O]^+^
						515	[M+HCO_3_^−^]^−^

RDA: Retro Diels-Alder reaction.

**Table 2:** The data on calibration curve, LODs and LOQs for 10 compounds.

Compound	Calibration Curve	R2	Linear Range (μg/mL)	LOD (μg/mL)	LOQ (μg/mL)
C1	y = 1890.4x + 44.73	R^2^ = 0.9990	6.6–264.0	0.15	0.51
C2	y = 2290.6x + 103.91	R^2^ = 0.9984	8.5–340.0	0.10	0.37
C3	y = 854.88x + 103.09	R^2^ = 0.9990	8.8–352.0	0.08	0.31
C4	y = 2663.5x − 9.4024	R^2^ = 0.9992	2.2–88.0	0.12	0.40
C5	y = 1310.9x − 16.697	R^2^ = 0.9995	3.7–148.0	0.08	0.35
C6	y = 4345x − 7.3054	R^2^ = 0.9994	1–40.0	0.06	0.26
C7	y = 5036.4x + 6.8332	R^2^ = 1.0000	5.1–204.0	0.09	0.35
C8	y = 3906.4x + 24.383	R^2^ = 0.9997	5.8–232.0	0.12	0.40
C9	y = 822.74x - 2.8707	R^2^ = 0.9996	6–240.0	0.04	0.18
C10	y = 2950.4x − 9.13	R^2^ = 0.9997	3.1–128.0	0.08	0.38

**Table 3:** Precision, stability, repeatability and recovery of 10 compounds.

Compound	Precision (n = 6)		Repeatability (n = 6)	Stability (n = 6)	Recovery (n = 5)	
	Intra-Day RSD (%)	Inter-Day RSD (%)	RSD (%)	RSD (%)	Average Recovery (%)	RSD (%)
C1	1.14	1.09	0.92	0.98	99.72	2.90
C2	1.29	0.76	0.8	1.29	98.94	2.51
C3	0.79	0.62	1.09	0.78	99.87	1.38
C4	1.49	1.32	1.24	1.01	100.24	1.71
C5	1.28	1.50	2.05	2.23	100.01	1.67
C6	1.22	2.02	2.63	2.27	99.83	1.58
C7	0.57	0.91	1.01	0.86	99.62	1.08
C8	0.41	0.52	0.31	0.44	100.12	0.49
C9	1.21	0.73	1.34	0.95	100.08	0.99
C10	0.54	1.13	1.33	1.08	100.57	1.47

**Table 4:** Content of 10 components in Phellodendri Amurensis Cortex (PAC) samples collected from different origins (mg/g).

No.	C1	RSD (%)	C2	RSD (%)	C3	RSD (%)	C4	RSD (%)	C5	RSD (%)	C6	RSD (%)	C7	RSD (%)	C8	RSD (%)	C9	RSD (%)	C10	RSD (%)	Similarity
S1	2.486	0.98	14.67	0.87	11.838	1.11	1.981	0.57	1.357	0.73	0.346	1.02	4.196	0.41	7.198	0.82	4.683	1.31	3.835	0.62	0.982
S2	3.423	1.02	6.543	1.21	5.935	0.87	3.422	0.86	0.636	0.39	0.299	1.67	1.756	1.38	3.59	0.44	5.793	1.09	4.368	0.81	0.896
S3	2.203	1.23	3.347	1.14	1.47	1.38	2.795	1.32	0.416	1.21	0.199	0.96	0.62	1.12	1.935	0.79	3.827	1.45	3.389	1.07	0.791
S4	3.249	0.83	5.446	0.88	5.534	0.96	2.171	2.19	0.819	2.39	0.236	2.08	2.744	0.75	3.923	0.68	5.578	0.82	2.432	1.18	0.968
S5	4.448	1.57	8.695	1.47	6.75	1.21	2.129	1.44	1.276	1.76	0.181	2.19	2.966	0.89	3.842	1.04	5.915	1.25	1.621	0.77	0.972
S6	2.035	1.89	5.876	1.36	4.54	1.76	0.312	1.61	1.392	0.93	0.539	0.49	4.945	1.31	6.138	2.02	7.25	0.81	2.927	1.41	0.951
S7	3.442	1.45	6.503	0.99	7.242	0.78	0.348	0.65	1.367	0.55	0.317	1.25	5.483	1.08	4.355	1.06	5.466	0.3	2.561	1.3	0.933
S8	2.324	2.12	3.679	1.98	4.92	1.68	0.331	0.29	1.036	0.61	0.198	0.29	3.84	1.47	2.541	2.09	5.662	2.71	2.348	1.22	0.895
S9	1.135	1.12	5.681	1.27	6.409	1.31	1.586	1.32	1.106	1.18	0.21	0.78	2.817	2.07	4.259	2.13	2.48	1.41	2.93	1.44	0.974
S10	1.525	0.97	7.507	1.08	7.816	1.19	0.907	1.49	1.06	2.28	0.319	0.81	3.994	0.69	2.789	1.63	5.364	1.67	1.128	0.78	0.941
S11	2.328	0.89	7.631	0.69	4.687	0.75	0.794	0.78	0.956	1.83	0.221	1.28	2.709	0.58	4.424	1.09	3.636	1.45	1.289	0.38	0.997
S12	3.409	1.24	14.579	1.17	20.893	1.48	1.415	0.48	1.668	0.91	0.398	1.48	2.15	0.75	8.184	0.88	8.37	2.39	4.312	0.45	0.945
S13	3.055	1.45	19.561	1.59	16.133	1.29	1.029	0.74	3.286	1.15	0.648	0.82	8.75	1.27	14.902	0.74	7.381	2.04	4.568	1.06	0.983
S14	3.767	2.01	17.004	1.87	11.247	1.79	3.038	1.61	2.022	0.86	0.527	1.36	7.346	0.38	6.965	1.22	9.781	0.94	3.883	1.72	0.966
S15	3.732	0.78	18.027	0.47	18.068	0.91	0.659	2.39	2.283	1.23	0.258	1.27	3.363	1.51	12.096	0.93	12.065	1.31	1.952	1.65	0.972
S16	4.214	0.76	11.433	0.87	7.66	1.01	0.953	1.38	1.885	0.88	0.338	0.79	3.734	1.81	10.399	0.84	10.314	1.38	3.116	1.27	0.981
S17	2.257	0.85	12.215	1.26	10.822	1.17	1.488	0.88	1.425	1.33	0.519	1.21	2.614	0.81	11.304	1.28	13.042	0.35	2.638	0.89	0.959
S18	1.688	1.41	10.82	1.98	16.053	1.39	2.679	1.41	1.119	2.67	0.414	2.11	3.36	0.91	8.877	0.79	9.013	0.56	1.764	1.21	0.979
S19	3.498	1.78	12.776	1.47	6.336	2.01	1.341	2.09	0.768	0.83	0.295	0.73	2.509	1.29	7.584	1.23	8.098	0.48	2.738	1.43	0.976
S20	3.88	2.03	15.232	2.01	14.196	1.87	2.57	2.31	1.526	0.76	0.401	1.56	2.797	1.34	11.262	1.48	11.352	0.84	3.084	2.31	0.969
S21	3.042	2.45	12.624	2.27	17.622	1.93	1.597	0.99	1.956	0.37	0.273	0.47	4.413	0.81	10.629	0.75	9.858	1.71	2.897	1.76	0.988
S22	4.976	1.67	19.047	1.87	18.119	1.54	2.419	0.87	3.2	0.36	0.558	1.13	12.756	0.24	9.425	0.81	7.684	1.25	1.48	1.24	0.981
S23	3.176	0.59	14.27	0.91	12.593	0.71	0.956	0.31	1.895	1.28	0.418	1.19	3.466	0.86	10.76	1.21	11.854	0.58	3.115	1.42	0.937
S24	9.939	1.71	27.521	1.39	11.511	1.23	0.895	0.84	3.355	2.29	0.373	1.42	8.969	0.43	19.931	1.49	5.03	0.83	0.727	0.65	0.986
S25	4.37	0.91	27.438	0.8	21.721	0.57	0.551	1.18	2.808	2.15	0.743	1.45	6.041	1.33	20.287	2.01	6.546	0.59	2.646	0.75	0.975
S26	10.135	0.76	21.581	0.58	15.515	1.12	1.59	1.27	2.492	1.31	0.614	2.19	11.67	2.02	11.11	1.49	5.193	1.26	1.133	1.23	0.963
S27	3.35	1.23	21.115	1.53	18.304	0.99	1.588	1.49	2.93	1.46	0.346	1.18	8.241	1.12	13.09	1.28	3.371	1.49	2.282	1.19	0.993
S28	5.104	2.22	19.523	2.08	21.509	1.84	1.443	1.39	2.837	0.31	0.379	1.13	9.34	0.37	9.646	2.01	4.319	1.67	1.132	1.08	0.972
S29	16.227	0.98	17.129	0.69	7.187	0.76	2.218	0.28	2.864	0.39	0.61	1.21	9.895	0.77	19.015	0.58	6.12	2.03	2.027	2.03	0.959
S30	14.082	0.84	19.853	0.64	12.562	0.94	1.378	0.58	2.815	0.44	0.596	1.27	10.054	1.37	13.117	0.69	5.123	1.21	1.045	1.34	0.973
S31	2.564	0.46	17.129	0.86	16.921	0.84	1.436	0.46	2.053	0.69	0.33	2.37	6.286	2.24	10.162	0.96	4.018	1.04	0.977	0.95	0.991
S32	1.829	1.13	19.971	1.4	14.767	1.62	1.03	0.35	3.182	0.65	0.531	0.84	4.923	2.12	16.661	0.81	2.352	0.97	0.444	0.45	0.967
S33	9.665	1.34	29.666	1.2	13.621	1.71	1.051	0.93	4.17	1.34	0.673	1.67	9.091	1.03	25.745	0.92	6.226	0.38	1.199	0.49	0.975

C1:3-O-feruloylquinic acid; C2: 4-O-feruloylquinic acid; C3: magnoflorine; C4: syringing; C5: phellodendrine; C6: jatrorrhizine; C7: palmatine; C8: berberine; C9: obaculactone; C10: obacunone.

**Table 5:** Samples from different locations.

No.	Geographical Location
S1–4	Yichun, Heilongjiang
S5–8	Wuchang, Heilongjiang
S9–11	Mudanjiang, Heilongjiang
S12–14	Yanbian, Jilin
S15–16	Tonghua, Jilin
S17–19	Jiaohe, Jilin
S20–23	Huadian, Jilin
S24–26	Fengcheng, Liaoning
S27–30	Anshan, Liaoning
S31–33	Zhuanghe, Liaoning
